# Spiritual care competence and its predictors among critical and emergency nurses: a cross-sectional study of spiritual intelligence and death attitudes

**DOI:** 10.1016/j.ijnsa.2026.100596

**Published:** 2026-06-09

**Authors:** Sara Rahimi, Mojtaba Senmar, Mohammad Mozafari, Mahdie Bahrami, Rahman Panahi, Marzieh Khatooni

**Affiliations:** a Social Determinants of Health Research Center, Research Institute for Prevention of Non-Communicable Diseases, Qazvin University of Medical Sciences, Qazvin, Iran; b Non-communicable Diseases Research Center, Research Institute for Prevention of Non-Communicable Diseases, Qazvin University of Medical Sciences, Qazvin, Iran; c Student Research Committee, Qazvin University of Medical Sciences, Qazvin, Iran; d Health Metrics Research Centre, Iranian Institute for Health Sciences Research, ACECR, Tehran, Iran

**Keywords:** Spiritual care competence, Spiritual intelligence, Attitude to death, Critical care nursing, Emergency nursing, Holistic nursing

## Abstract

**Background:**

Spiritual care is a core element of holistic nursing, especially in demanding settings such as emergency and intensive care units where patients often face life-threatening conditions and end-of-life challenges. Despite its importance, little is known about nurses’ competence in providing spiritual care and the factors that shape it.

**Objective:**

This study aimed to explore spiritual care competence and its predictors among nurses working in critical and emergency settings.

**Design:**

A cross-sectional study.

**Settings:**

The study was conducted in the emergency and intensive care units of two central hospitals in Qazvin, Iran.

**Participants:**

A total of 180 nurses working in critical care and emergency units participated in the study.

**Methods:**

Data were collected using the Spiritual Care Competence Scale, the Spiritual Intelligence Self-Report Inventory, and the Death Attitude Profile-Revised. Descriptive statistics and logistic regression analyses were performed using SPSS version 23.

**Results:**

Nurses demonstrated moderate levels of spiritual care competence (86.79 ± 4.25) and spiritual intelligence (57.15 ± 6.13). Among death attitude dimensions, neutral acceptance scored highest, while approach acceptance scored lowest. Logistic regression revealed that age, work experience, spiritual intelligence, and a positive attitude toward death were significant predictors of spiritual care competence (p < 0.05).

**Conclusion:**

Spiritual care competence among nurses is associated with personal maturity, professional experience, spiritual intelligence, and positive attitudes toward death. Integrating spiritual care education and fostering positive perspectives on death may enhance nurses’ preparedness to deliver comprehensive, patient-centered care.

**What is already known**

• Spiritual care is a recognised component of holistic nursing, particularly in emergency and intensive care settings.
• Spiritual intelligence and attitudes toward death have each been linked to aspects of professional competence in nursing.
• Nurses in high-acuity environments often report moderate levels of spiritual care competence.

Alt-text: Unlabelled box dummy alt text

**What this paper adds**

• This study examines spiritual intelligence and attitudes toward death together as predictors of spiritual care competence among emergency and intensive care unit nurses.
• Spiritual intelligence and positive attitudes toward death were independently associated with higher odds of optimal spiritual care competence in multivariable analysis.
• The findings provide context-specific evidence from high-acuity hospital settings and inform future educational development.

Alt-text: Unlabelled box dummy alt text

## 1 Introduction

Healthcare professionals bear the primary responsibility for delivering comprehensive, holistic care that encompasses the physical, psychological, emotional, spiritual, and social dimensions of patients, while simultaneously preventing potential harm (Booth and Kaylor, 2018; Riahi et al., 2018). Within this framework, spiritual care has been recognized as a fundamental component of nursing practice, defined as the systematic assessment of and response to patients' spiritual needs with the aim of enhancing spiritual well-being and promoting overall functioning (Machul et al., 2022). Patients confronting severe physical, psychological, emotional, or social challenges are particularly vulnerable and in need of targeted spiritual care (Pierce et al., 2020), which is considered an integral dimension of nursing practice in settings characterized by critical illness or end-of-life care (Riahi et al., 2018). The provision of spiritual care has been empirically associated with improved quality of life (Chen et al., 2018), reduced anxiety (Salari et al., 2023), and enhanced spiritual health (Connolly and Timmins, 2021). In end-of-life contexts, spiritual care has been shown to further improve quality of life and patients' sense of worth (Khorrami et al., 2020; Inaloo et al., 2025), as well as to alleviate death-related anxiety (Inaloo et al., 2025). Despite these documented benefits, the factors that contribute to nurses’ ability to competently deliver spiritual care, particularly in high-acuity settings such as emergency and intensive care units, remain insufficiently clarified.

## 2 Background

The effective delivery of spiritual care requires specific professional skills, including active listening, competence in spiritual assessment, and the ability to integrate spiritual considerations into comprehensive patient care (Asgari et al., 2022). These skills are encompassed within the construct of spiritual care competence, defined as the integrated knowledge, skills, and attitudes necessary to assess patients' spiritual needs and implement effective interventions (Cao et al., 2020; Daghan et al., 2019). Van Leeuwen and Cusveller identified three core domains of spiritual care competence: self-awareness and self-use, integration of spirituality into the nursing process, and quality assurance (Van Leeuwen et al., 2009). In culturally and religiously oriented societies such as Iran, where spirituality occupies a central role, strengthening and evaluating nurses’ spiritual care competence is particularly important for ensuring care quality (Heidari et al., 2022). Nurses working in intensive care and emergency units face heavy workloads, frequent exposure to death and dying, rapid clinical decision-making demands, and sustained responsibility for critically ill patients (Bolado et al., 2024; Han et al., 2023). In such environments, patients’ spiritual needs may be unintentionally overlooked (Dallı, 2025), underscoring the need to identify factors associated with higher levels of spiritual care competence.

Among the factors potentially related to spiritual care competence is spiritual intelligence, a construct integrating elements of intelligence and spirituality and referring to the capacity to draw upon spiritual and religious resources for adaptive coping, problem-solving, and humane decision-making (Abdorazaghnejad et al., 2020; Sharifnia et al., 2022). Attributes such as faith, humility, gratitude, emotional regulation, morality, forgiveness, and compassion are considered hallmarks of spiritual intelligence (Riahi et al., 2018). Higher levels of spiritual intelligence have been associated with greater meaning in professional roles, improved coping with occupational stressors, and reduced stress among healthcare professionals (Arad et al., 2020). In religious societies such as Iran, spiritual intelligence may also strengthen nurse–patient relationships and enhance overall care quality (Arad et al., 2020; Emamgholian et al., 2017). Moreover, spiritual intelligence involves the ability to understand and manage one’s own emotions and those of others, thereby fostering positive interpersonal relationships and professional growth (Emmons, 2000). Variations in nurses’ levels of spiritual intelligence have also been linked to differences in attitudes toward death (Kudubes et al., 2021; Riahi et al., 2018). Nevertheless, evidence suggests that the spiritual intelligence of Iranian nurses has often been reported at moderate or low levels (Abdorazaghnejad et al., 2020; Goudarzi et al., 2019; Moradnezhad et al., 2017).

Nurses frequently encounter death in clinical practice, and caring for dying patients and supporting their families represents one of the most challenging aspects of nursing. Nurses’ attitudes toward death can influence care quality, the expression of compassion, and the support provided during end-of-life decision-making (Goyarrola et al., 2024; Kudubes et al., 2021). Attitude toward death refers to the cognitive, emotional, and behavioral responses individuals hold regarding the reality of death (Ahmadi Farsani et al., 2019). These attitudes may be characterized by fear or avoidance, or by acceptance and adaptive coping (Duran and Polat, 2024). They are shaped by cultural context, beliefs, personal experiences, age, gender, and socioeconomic factors (Glushich et al., 2025). Empirical findings indicate that a more accepting attitude toward death is associated with improved quality of care (Schroeder and Lorenz, 2018; Wang et al., 2018), whereas negative attitudes may hinder effective spiritual care and compromise the recognition of patients’ spiritual needs (Li et al., 2021).

## 3 Purpose of the study

Given the central importance of spiritual care in holistic nursing practice and the specific challenges of emergency and intensive care environments, the present study aimed to examine predictors of Spiritual Care Competence among nurses working in these settings. Specifically, this study investigated the relationships between spiritual intelligence, attitudes toward death, and selected demographic characteristics with spiritual care competence. While prior studies have examined these variables separately, their combined examination within high-acuity emergency and intensive care contexts remains limited. By focusing on emergency and intensive care nurses in Iran, this study provides context-specific evidence regarding factors associated with spiritual care competence in high-acuity clinical settings, where exposure to critical illness and death is routine and spiritual needs may be more complex. The findings may inform future educational and organizational strategies designed to strengthen nurses’ ability to address patients’ spiritual needs effectively.

## 4 Methods

### 4.1 Study design

This cross-sectional study was conducted between 2024 and 2025 among nurses working in the adult emergency and intensive care units of two hospitals affiliated with Qazvin University of Medical Sciences. The reporting of this study was conducted in accordance with the STROBE (Strengthening the Reporting of Observational Studies in Epidemiology) guidelines for cross-sectional studies.

### 4.2 Setting and participants

Nurses with at least one year of work experience and a bachelor’s degree or higher were eligible. The requirement of a minimum of one year of clinical experience was applied to ensure adequate exposure to emergency and intensive care environments, thereby enabling an appropriate evaluation of nurses’ professional competence, consistent with criteria commonly adopted in nursing competency research (Jafari Rahim Abad et al., 2025). Sampling was conducted using a stratified approach based on hospital and clinical unit (emergency department and intensive care units), with each hospital-unit combination constituting a distinct stratum. Eligible nurses were identified from staff rosters within each stratum. Staff rosters were obtained from the nursing management offices of each hospital unit. To achieve the required sample size, all eligible nurses across the defined strata were invited to participate, and recruitment continued until the target was reached. Participants were recruited through in-person visits to the emergency and intensive care units by the research team. The purpose of the study was explained to eligible nurses, and those who consented received the study questionnaires. Participation was voluntary. The planned sample size was determined based on established recommendations for multivariable regression analysis, suggesting a minimum of 10–15 observations per predictor variable to obtain stable estimates (Hosmer Jr et al., 2013). Given the inclusion of 11 independent variables, a minimum sample of approximately 165 participants was considered adequate. In addition, evidence suggests that the traditional 10 events-per-variable rule for logistic regression may be overly conservative in applied research settings (Vittinghoff and McCulloch, 2007). To enhance statistical stability and account for potential non-participation or incomplete responses, the final target sample was set at 180 nurses, which was fully achieved.

### 4.3 Measures

#### 4.3.1 Section 1: demographic data

Demographic data were collected using a researcher-designed checklist, which included age, gender, marital status, education level, economic status, work experience, type of employment, and work shift.

#### 4.3.2 Section 2: spiritual care competence scale

Spiritual care competence was assessed using the 27-item spiritual care competence scale developed by van Leeuwen (2009). This instrument consists of six dimensions: assessment and implementation of spiritual care, professionalization and improvement of spiritual care quality, personal support and patient counseling, referral to specialists, attitudes toward patients’ spirituality, and communication. Responses were rated on a five-point Likert scale, with a total score ranging from 27 to 135; higher scores indicate greater competence (Van Leeuwen et al., 2009). In the present study, and in line with previous research, spiritual care competence was categorized into two levels: limited competence (scores 27–62) and desirable competence (scores 63–135) (Sahebi and Barkhordari-Sharifabad, 2023), which were subsequently used in logistic regression analyses. The spiritual care competence scale has been widely disseminated and psychometrically evaluated across diverse cultural contexts, with recent reviews confirming its robust measurement properties (van Leeuwen and Schep-Akkerman, 2024). Previous studies have confirmed the validity and reliability of this questionnaire for use in the Iranian context (Khalaj et al., 2013). In the present study, the internal consistency of the spiritual care competence scale was acceptable, with a Cronbach’s alpha of 0.87.

#### 4.3.3 Section 3: spiritual intelligence self-report inventory

Spiritual Intelligence Self-Report Inventory, developed by King and DeCicco (2009), is a 24-item self-report measure assessing four dimensions of spiritual intelligence: critical existential thinking, personal meaning production, transcendental awareness, and conscious state expansion. Items are rated on a 5-point Likert scale (0 = completely wrong to 4 = completely right), with item 6 scored in reverse. Total scores range from 0 to 96; scores below 32 indicate low, 32–64 moderate, and above 64 high spiritual intelligence. In the original English version, Cronbach’s alpha was 0.92 for the total scale and 0.78–0.91 for the subscales (King and DeCicco, 2009). This instrument has also been psychometrically validated in Iranian populations, supporting its reliability and suitability for use in local research settings (Raghib et al., 2010). In the present study, the Cronbach’s alpha for the total spiritual intelligence self-Report inventory score was 0.91, indicating good internal consistency.

#### 4.3.4 Section 4: death attitude profile-revised

The Death Attitude Profile-Revised, developed by Wong, Reker, and Gesser in 1994, consists of 32 items across five dimensions of attitudes toward death: fear of death, death avoidance, neutral acceptance, approach acceptance, and escape acceptance. According to the conceptual framework of the instrument, “Fear of Death” and “Death Avoidance” represent negative attitudes toward death, whereas “Neutral Acceptance”, “Approach Acceptance”, and “Escape Acceptance” reflect positive attitudes toward death. Items are rated on a seven-point Likert scale (1 = strongly disagree to 7 = strongly agree), and the mean score for each subscale is calculated by dividing the total subscale score by the number of items. The total score ranges from 32 to 224, with higher scores in each subscale indicating a stronger endorsement of that specific attitude dimension (Wong et al., 1994). The applicability of this instrument for the Iranian population has been verified (Haratiyan et al., 2019). In this study, the total death attitude profile-revised demonstrated a Cronbach’s alpha coefficient of 0.92, reflecting strong internal consistency.

### 4.4 Data collection

Data were collected using self-administered paper-based questionnaires distributed in person by a trained research assistant during working shifts. All eligible nurses in the selected units were approached and invited to participate. Questionnaires were provided to those who consented and were completed anonymously during work breaks or less busy periods of the shift. Completed questionnaires were returned in sealed envelopes to a designated collection box within each unit. To enhance participation and ensure adequate coverage of different shifts, the distribution and collection process was conducted across multiple working shifts. When necessary, brief reminders were provided. In addition, questionnaires were reviewed at the time of collection to ensure completeness, which minimized missing data. As a result of this structured in-person administration, all distributed questionnaires were returned without missing responses. As all distributed questionnaires were returned complete, there were no missing data in the analyzed sample; therefore, non-response bias was not considered a concern. Similarly, as data collection was conducted within a single, relatively short time frame, a comparison between early and late responders was not methodologically meaningful. To reduce potential social desirability and common method bias, participants were assured that their responses would remain confidential, would not affect their employment status, and would be used solely for research purposes. No identifying information was collected.

### 4.5 Data analysis

Data were entered into SPSS version 23 and analyzed using descriptive statistics and logistic regression. In this study, spiritual care competence was considered the dependent variable, while age, gender, marital status, educational level, economic status, work experience, employment type, work shift, spiritual intelligence, and positive and negative attitudes toward death were included as independent variables. Age and work experience were entered into the regression model as categorical variables based on predefined groupings (25–34, 35–44, and 45–54 years for age; <10, 10–20, and >20 years for work experience). The first category of each categorical variable was treated as the reference group, and independent variables were entered simultaneously using the Enter method, with categorical predictors specified as indicator (dummy) variables, and the first category selected as the reference class. Spiritual intelligence and positive and negative attitudes toward death were entered as continuous variables using their total scores. A significance level of *p* < 0.05 was considered for all analyses.

Spiritual care competence is originally classified into three levels (low, moderate, and high) according to the instrument scoring guidelines. For the purpose of regression analysis, and in line with prior empirical studies conducted in the Iranian context (Sahebi and Barkhordari-Sharifabad, 2023), the outcome variable was dichotomized by defining the low category as limited competence and combining the moderate and high categories as desirable (optimal) competence. Although this approach facilitated logistic regression modeling and interpretation, the potential loss of information associated with dichotomization is acknowledged as a methodological limitation. This decision was made to align the analysis with a clinically interpretable distinction between insufficient and acceptable competence levels.

Before interpreting the regression results, model diagnostics were performed. The Hosmer–Lemeshow test was used to assess goodness of fit, and the results indicated an adequate model fit (df = 8, *P* = 0.187, χ² = 11.273). Nagelkerke’s R² was calculated to estimate the explanatory power of the model, yielding a value of 0.125, which indicates that approximately 12.5% of the variance in spiritual care competence was explained by the predictors included in the model. To address the potential risk of common method bias, several procedural safeguards were employed during data collection: validated and psychometrically robust instruments were used, responses were collected anonymously, and participants were assured of confidentiality to reduce social desirability and evaluation apprehension. These steps were taken to reduce the likelihood of common method variance inherent in self-report survey research. Multicollinearity among continuous predictors was examined using Pearson correlation analysis. Correlation coefficients among spiritual intelligence, positive attitude, and negative attitude were all below 0.30, indicating low intercorrelations. The categorization of age and work experience was based on the original data collection format, as these variables were initially designed and recorded in grouped categories.

### 4.6 Ethical considerations

This study was approved by the Institutional Review Board of Qazvin University of Medical Sciences (Approval Code: IR.QUMS.REC.1402.297) and was conducted in full accordance with the ethical principles of the Declaration of Helsinki and its subsequent revisions. Prior to data collection, the purpose of the study, the procedures involved, and the voluntary nature of participation were explained to all eligible nurses. Written informed consent was obtained from each participant before enrollment. Participants were explicitly informed of their right to withdraw from the study at any time without any consequences for their employment status or clinical duties. No financial or material incentives were offered for participation. All data were collected anonymously; no personally identifying information was recorded. Questionnaires were completed in sealed envelopes returned to a designated collection box, and data were stored securely and used exclusively for research purposes. To minimize the risk of social desirability bias and evaluation apprehension, participants were assured that their responses were entirely confidential and would have no bearing on their professional standing.

## 5 Results

A total of 180 nurses participated in this study, representing the complete target sample across all defined strata. Of these participants, 43.9% (*n* = 79) were aged 35–44 years, 67.8% (*n* = 122) were female, and 56.1% (*n* = 101) were single. Additional demographic and professional characteristics of the nurses are summarized in Table 1.

**Table 1:** Frequency distribution of the nurses in the study by demographic and background variables (N = 180).

Variable	Levels	Frequency	Percentage
Age	25–34 years	61	33.9%
	35–44 years	79	43.9%
	45–54 years	40	22.2%
Gender	Male	58	32.2%
	Female	122	67.8%
Marital status	Single	101	56.1%
	Married	79	43.9%
Education level	Bachelor's	142	78.9%
	Higher than Bachelor's	38	21.1%
Economic status	Average	54	30.0%
	Good	81	45.0%
	Excellent	45	25.0%
Work experience	<10 years	56	31.1%
	10–20 years	77	42.8%
	>20 years	47	26.1%
Employment type	Contract	45	25.0%
	Permanent	135	75.0%
Work shift	Morning	72	40.0%
	Evening	108	60.0%

The mean score of spiritual care competence among the nurses was 86.79 ± 4.258, corresponding to a moderate level of competence based on the instrument’s scoring framework. According to the original three-level classification of the scale, participants were categorized as having low, moderate, or high competence. In the present sample, 19.5% (*n* = 35) were classified as having limited competence (low level), while 80.5% (*n* = 145) fell within the moderate or high competence categories. The mean score of spiritual intelligence was 57.15 ± 6.13, also reflecting a moderate level among the participants.

Table 2 presents the mean scores and standard deviations for the five dimensions of death attitudes. Among these dimensions, the highest scores were observed for “neutral acceptance” and “escape acceptance,” while “approach acceptance” received the lowest score.

**Table 2:** Mean and standard deviation of death attitude components among the nurses in the study (N = 180).

Variable	Item average ± SD	Range	Total score ± SD
Fear of death	4.30 ± 0.66	7–49	30.15 ± 2.87
Death avoidance	4.44 ± 0.59	5–35	23.22 ± 1.54
Neutral acceptance	5.15 ± 0.69	5–35	25.75 ± 3.15
Approach acceptance	3.94 ± 0.79	10–70	39.48 ± 5.08
Escape acceptance	4.71 ± 0.59	5–35	23.56 ± 2.57

Logistic regression analysis (Table 3) identified age, work experience, spiritual intelligence, and positive attitudes toward death as significant predictors of optimal spiritual care competence (*p* < 0.05). A clear gradient was observed across professional maturity variables: nurses aged 45–54 years had 1.41 times higher odds of demonstrating optimal competence compared with those aged 25–34 years, and those with >20 years of experience showed 1.33 times higher odds than nurses with <10 years of experience. These findings suggest a pattern in which greater age and accumulated clinical experience are associated with higher competence levels.

**Table 3:** Factors affecting optimal spiritual care competence among the nurses in the study based on logistic regression analysis^a^.

Variable	Levels	Odds ratio	Confidence interval (Lower–Upper)	*p*-value
Age				0.036*
	25–34 years	Reference		
	35–44 years	0.90	0.32–1.60	0.126
	45–54 years	1.41	1.01–2.02	0.029*
Gender				0.557
	Male	Reference		
	Female	0.56	0.18–1.01	0.414
Marital status				0.521
	Single	Reference		
	Married	0.50	0.10–1.06	0.616
Education level				0.259
	Bachelor	Reference		
	Higher than Bachelor	0.56	0.20–1.00	0.322
Economic status				0.244
	Moderate	Reference		
	Good	0. 66	0.31–1.10	0.489
	Excellent	1.15	0.26–2.18	0.425
Work experience				0.024*
	<10 years	Reference		
	10–20 years	0.58	0.12–1.11	0.142
	>20 years	1.33	1.21–1.55	0.011*
Employment type				0.547
	Contract	Reference		
	Staff	0.72	0.34–1.11	0.328
Shift				0.785
	Morning	Reference		
	Evening	0.79	0.42–1.26	0.361
Spiritual intelligence	-	2.21	1.19–3.39	**0.003**⁎⁎
Negative attitude	-	0.65	0.34–1.21	0.091
Positive attitude	-	1.75	1.57–2.22	**0.002**⁎⁎
Constant		2.10		0.112

(the number of participants included in the logistic regression analysis=180, Hosmer–Lemeshow test P-value=0.187, Nagelkerke’s R² = 0.125, overall percentage correct classification=55.67, Number of participants with “optimal” spiritual care competence= 145, total N = 180).

ᵃIndependent variables were entered simultaneously into the logistic regression model.

⁎ *p* < 0.05

⁎⁎ *p* < 0.001.

Spiritual intelligence demonstrated the strongest association in the model; each one-unit increase in score was linked to a 2.21-fold increase in the odds of optimal competence, indicating a substantial relationship between intrapersonal spiritual capacity and professional performance in spiritual care. Similarly, higher positive attitudes toward death were associated with increased likelihood of optimal competence (OR = 1.75), highlighting the relevance of cognitive-emotional perspectives on death in high-intensity clinical settings. Other demographic and professional variables were not significantly associated with competence (*P* > 0.05).

## 6 Discussion

This study examined the predictors of spiritual care competence among nurses working in emergency and intensive care settings, with a particular focus on age, work experience, spiritual intelligence, and death attitudes. Collectively, these four factors emerged as significant predictors of optimal spiritual care competence, a finding that carries meaningful implications for nursing education, workforce development, and clinical practice in high-acuity environments.

The mean competence score observed in the present study (86.79 ± 4.25) positioned nurses at a moderate level, broadly consistent with reports from Turkey (Dallı, 2025), Iran (Manookian et al., 2023), China (Li et al., 2021), and Malaysia (Abusafia et al., 2021). This cross-cultural convergence around a mid-range level suggests that moderate spiritual care competence is not an artifact of any single healthcare system but rather a widely shared baseline, highlighting the universal need for structured educational investment in this domain (Heidari et al., 2022).

Age was a significant positive predictor of competence, with nurses in the 45–54-year bracket being more likely to achieve optimal scores compared with younger colleagues. This pattern aligns with evidence from Machul et al. (2022) and Semerci et al. (2021), who similarly observed higher spiritual care competence among older nurses, and with Markani et al. (2018), who attributed this advantage to accumulated clinical exposure, broader encounter with existential patient concerns, and greater personal maturity. The relationship is not, however, linear or universal. Seid and Abdo (2022) reported an age-related decline in competence, and Alshakhshir et al. (2025) found that nurses over 50 scored lower than mid-career colleagues, possibly because the latter benefited from more recent curricular reforms. Together, these inconsistencies point to age as a proxy for other mediating variables, such as years of experience, educational background, and cultural context, rather than an independent determinant. Future research should disentangle these correlated constructs before drawing programmatic conclusions.

Work experience independently predicted competence, reinforcing a pattern documented across multiple international studies (Alshakhshir et al., 2025; Han et al., 2023; Machul et al., 2022; Semerci et al., 2021). Experienced nurses bring to the bedside a richer repertoire of clinical encounters, more refined assessment skills, and, when supported by in-service training, greater confidence in addressing patients' spiritual needs (Han et al., 2023; Seid and Abdo, 2022). Nevertheless, experience alone does not guarantee competence. Several studies found no significant association (Heidari et al., 2022; Li et al., 2021), and inverse relationships have been observed in contexts where organisational cultures deprioritise spiritual care or where prolonged service fosters burnout and detachment (Bakar et al., 2017; Markani et al., 2018). These contradictions underscore that experience is a modifiable but context-dependent asset; targeted professional development pathways, rather than time in service per se, are likely to yield more consistent gains in competence (Rabiei Vaziri et al., 2025).

Spiritual intelligence emerged as a meaningful predictor of spiritual care competence, contributing to the study's core argument that intrinsic nurse-level resources shape care quality independently of structural variables. Although studies directly investigating this dyad remain scarce, the weight of available evidence points in a consistent direction. Emamgholian et al. (2017) demonstrated a positive association between spiritual intelligence and spiritual care competence among nursing students, while Ahmadi et al. (2021) found that higher spiritual intelligence was linked to enhanced professional competence and self-awareness. At a broader level, Sharifnia et al.'s (2022) systematic review confirmed that spiritual intelligence predicts clinical competence, professional performance, and personal commitment, dimensions that collectively undergird quality spiritual care. Crucially, spiritual intelligence appears amenable to structured training: intervention studies by Riahi et al. (2018) and Sharifnia et al. (2022) found that targeted programmes enhanced both spiritual care competence and occupational well-being, suggesting that investment in this domain yields returns beyond the spiritual care encounter itself. That nurses in the present study scored at only a moderate level on spiritual intelligence, consistent with Bazghaleh et al. (2023) and Mehralian et al. (2023) but below findings from higher-resource contexts (Kaur et al., 2015; Khosravi et al., 2022), reinforces the case for integrating spiritual intelligence training into nursing curricula, particularly in emergency and intensive care programmes where the emotional and existential demands of practice are greatest.

A positive attitude toward death was the final significant predictor identified in this study. Nurses scored highest on the neutral acceptance subscale and lowest on approach acceptance, replicating the pattern reported by Li et al. (2021). This configuration is consequential: Lee and Park (2025) identified attitudes toward end-of-life care (beta coefficient = 0.39) as the most influential predictor of spiritual care competence in their sample, accounting for 36.9% of the variance. Prior work corroborates a coherent explanatory mechanism, in that nurses who perceive death as a natural and meaningful event are more attuned to patients' existential distress and more willing to engage with it therapeutically (Li et al., 2021). Conversely, death avoidance and anxiety have been consistently associated with lower spiritual competence and more defensive clinical behaviour (Duran and Polat, 2024; Kudubes et al., 2021). The relevance of these dynamics is amplified in emergency and intensive care settings, where encounters with mortality are routine. Evidence suggests that nurses with a reflective orientation toward death demonstrate more supportive, ethically grounded responses to end-of-life decision-making (Kudubes et al., 2021; Kang and Han, 2013), and that structured education in palliative principles and death literacy strengthens both attitudes and downstream care quality (Chua and Shorey, 2021; Lo Iacono et al., 2024). Some findings do temper this narrative; Tüzer et al. (2020) found only a weak association between death attitudes and perceived spiritual care, suggesting that the relationship may be moderated by institutional culture or measurement approach and warrants further investigation.

Overall, the present findings underscore that spiritual care competence in high-acuity settings appears to be linked to both experiential maturity and existential–reflective capacities. Rather than attributing competence to any single factor, the results support a multidimensional perspective in which professional exposure, spiritual intelligence, and attitudes toward death collectively relate to nurses’ preparedness to address patients’ spiritual needs.

## 7 Limitations

This study has several limitations. First, its cross-sectional design precludes causal inferences between spiritual intelligence, attitudes toward death, and spiritual care competence. Second, the use of self-reported measures may introduce response biases, including social desirability, particularly in assessing constructs such as spiritual care competence, spiritual intelligence, and attitudes toward death, which may be influenced by professional expectations and cultural norms. Spiritual care competence, originally measured as a continuous three-level construct, was dichotomized for regression analysis, which may have reduced variability and resulted in some loss of information. In addition, preliminary group comparison analyses (for example, *t*-tests or analysis of variance) were not conducted, potentially limiting descriptive insight into subgroup differences.

The sample was drawn from nurses working in emergency and intensive care units in two hospitals, which may limit representativeness and restrict generalizability to broader nursing populations or other clinical and cultural settings. Finally, as spiritual care competence is multifactorial, not all potentially relevant variables could be examined within a single study.

## 8 Conclusion

In conclusion, age, work experience, spiritual intelligence, and positive attitudes toward death were significantly associated with higher odds of optimal spiritual care competence among nurses working in emergency and intensive care units. Nurses with lower levels of these characteristics demonstrated reduced odds of desirable competence. These findings highlight meaningful associations between both professional experience and individual psychological resources and spiritual care competence in high-acuity settings. Given the cross-sectional design, the observed relationships should not be interpreted as causal. Moreover, the dichotomization of spiritual care competence for logistic regression analysis may have reduced variability and limited analytical precision. Within these methodological boundaries, the study contributes to a more nuanced understanding of factors associated with spiritual care competence in critical care environments.

## 9 Implications for practice and research

Clinically, integrating structured training in spiritual care and nurse empowerment programs, particularly in high-stress settings, is essential to enhance holistic patient care. From a research perspective, future studies should employ longitudinal and mixed-methods designs and include culturally and clinically diverse samples to fully explore the relationships among spiritual intelligence, attitudes toward death, and spiritual care competence. Such evidence can guide the development of effective frameworks and interventions in nursing practice.

## Ethics approval and consent to participate

This study was approved by the Ethics Committee of Qazvin University of Medical Sciences (Approval Code: IR.QUMS.REC.1402.297). All participants provided written informed consent prior to participation. The study adhered to the principles of the Declaration of Helsinki and its subsequent revisions.

## Consent for publication

Not applicable.

## Funding

The authors gratefully acknowledge the Vice Chancellor for Research and Technology of Qazvin University of Medical Sciences for providing the financial support and essential resources necessary for conducting this research.

## CRediT authorship contribution statement

**Sara Rahimi:** Writing – original draft, Investigation, Data curation, Conceptualization. **Mojtaba Senmar:** Supervision, Resources, Formal analysis, Conceptualization. **Mohammad Mozafari:** Software, Investigation, Data curation, Conceptualization. **Mahdie Bahrami:** Software, Project administration, Investigation, Formal analysis, Conceptualization. **Rahman Panahi:** Writing – review & editing, Validation, Software, Resources, Methodology, Formal analysis. **Marzieh Khatooni:** Writing – review & editing, Supervision, Investigation, Conceptualization.

## Declaration of competing interest

The authors declare that they have no known competing financial interests or personal relationships that could have appeared to influence the work reported in this paper.

## Data Availability

The data that support the findings of this study are available from the corresponding author upon reasonable request.

## Appendix Supplementary materials

Image, application 1
