# Supplementary material for: Spiritual care competence and its predictors among critical and emergency nurses: a cross-sectional study of spiritual intelligence and death attitudes
Source: Int J Nurs Stud Adv. 2026 Jun 9;11:100596. doi: 10.1016/j.ijnsa.2026.100596 (PMC13292470; doi:10.1016/j.ijnsa.2026.100596)
Supplement: Supplementary file 1 [file mmc1.docx]

**Supplementary Table S1. Operational Definition and Coding of Study Variables**

| Variable | Type | Coding / Measurement | Reference Category | Notes |
| --- | --- | --- | --- | --- |
| Age | Categorical | 1 = 25–34, 2 = 35–44, 3 = 45–54 | 25–34 years | Entered as dummy variables |
| Gender | Categorical | 0 = Male, 1 = Female | Male | Binary indicator coding |
| Marital status | Categorical | 1 = Single, 2 = Married | Single | Dummy variable |
| Education level | Categorical | 1 = Bachelor’s, 2 = Higher than Bachelor’s | Bachelor’s | Reference = lowest level |
| Economic status | Categorical | 1 = Moderate, 2 = Good, 3 = Excellent | Moderate | Ordinal coding in SPSS |
| Work experience | Categorical | 1 = <10 years, 2 = 10–20 years, 3 = >20 years | <10 years | Dummy-coded |
| Employment type | Categorical | 1 = Contract, 2 = Permanent | Contract | Binary indicator |
| Work shift | Categorical | 1 = Morning, 2 = Evening | Morning | Binary indicator |
| Spiritual intelligence | Continuous | Total score (scale-based) | — | Higher scores indicate higher spiritual intelligence |
| Positive attitude toward death | Continuous | Mean score of Neutral Acceptance, Approach Acceptance, and Escape Acceptance subscales | — | Higher scores indicate more positive attitude |
| Negative attitude toward death | Continuous | Mean score of Fear of Death and Death Avoidance subscales | — | Higher scores indicate more negative attitude |
| Spiritual care competence | Dependent variable | 1 = Low (limited competence), 2 = Moderate/High (optimal competence) | Low competence | Outcome used in logistic regression |
